# CeRNA Network Reveals the Circular RNA Characterization in Goat Ear Fibroblasts Reprogramming into Mammary Epithelial Cells

**DOI:** 10.3390/genes14101831

**Published:** 2023-09-22

**Authors:** Jam Zaheer Ahmed Sahito, Shan Deng, Liangshan Qin, Lianggui Xiao, Dandan Zhang, Ben Huang

**Affiliations:** 1 College of Animal Science and Technology, Guangxi University, Nanning 530004, China; sahitozaheer@hotmail.com (J.Z.A.S.); dengshan0420@163.com (S.D.); qinliangshanjohnson@gmail.com (L.Q.); gxuerxlg@163.com (L.X.); z18775306192@163.com (D.Z.); 2 Guangxi Key Laboratory of Eye Health, The People’s Hospital of Guangxi Zhuang Autonomous Region, Guangxi Academy of Medical Sciences, Nanning, 530021, China

**Keywords:** goat, circRNAs, CiMECs, ceRNAs network, RNA-seq

## Abstract

Circular RNAs (circRNAs) are a type of non-coding RNA that play a crucial role in the development and lactation of mammary glands in mammals. A total of 107 differentially expressed circRNAs (DE circRNAs) were found, of which 52 were up-regulated and 55 were down-regulated. We also found that DE circRNA host genes were mainly involved in GO terms related to the development process of mammary epithelial cells and KEGG pathways were mostly related to mammary epithelial cells, lactation, and gland development. Protein network analysis found that DE circRNAs can competitively bind to miRNAs as key circRNAs by constructing a circRNA–miRNA–mRNA network. CircRNAs competitively bind to miRNAs (miR-10b-3p, miR-671-5p, chi-miR-200c, chi-miR-378-3p, and chi-miR-30e-5p) involved in goat mammary gland development, mammary epithelial cells, and lactation, affecting the expression of core genes (*CDH2*, *MAPK1*, *ITGB1*, *CAMSAP2*, and *MAPKAPK5*). Here, we generated CiMECs and systematically explored the differences in the transcription profile for the first time using whole-transcriptome sequencing. We also analyzed the interaction among mRNA, miRNA, and cirRNA and predicted that circRNA plays an important role in the maintenance of mammary epithelial cells.

## 1. Introduction

As an effective new approach for biomedical research and regenerative medicine, direct reprogramming can dramatically transform one cellular identity into another [1]. To date, multiple methods have been used to reprogram somatic cells, including cellular factors; however, genetic manipulation in these reprogramming strategies may raise safety concerns. Small molecules are advantageous in reprogramming without gene manipulation because they are more cost-effective, simple to synthesize, stable, and can be standardized. The concentrations and combinations of small molecules can be altered to optimize the reprogramming strategy [2]. This method has been reported to permit lineage conversion between functional somatic cell types such as neuron, cardiomyocyte, hepatocyte-like, and mammary epithelial cells. Even though reprogramming cells and initial cells share the same genome, different gene expression patterns may define their identities, and sustaining a stable new lineage expression pattern is the key to reprogramming success. However, the identity maintenance mechanism of reprogrammed cell is not clear; therefore, it is necessary to further explore this unknown molecular mechanism.

Only a minuscule portion of each eukaryotic genome can encode proteins. In the past, “junk DNA” was used to describe DNA that was not encoded or of which the function was unknown. As sequencing technology advances, increasing evidence suggests that ncRNA plays a crucial role in various biological processes during development, particularly in the reprogramming mechanism. Non-coding RNA (ncRNA) is divided into long-chain non-coding RNA (lncRNA) based on its length (200 nt).

Long intergenic ncRNA (lincRNA) comprises cyclic RNA (circRNA), enhancer RNA (eRNA), antisense transcripts, and lincRNA. CircRNA is a circular RNA produced by reverse splicing of mRNA precursors. It is a key regulator in many biological processes and has been proven to be a “sponge” for miRNA, such as cirRS-7/CDR1as, which functions as a regulator of miR-7 [3]. Reprogramming involves energy and metabolism, and other researchers have demonstrated that mcPGK1 (a mitochondrial circRNA) can promote the mitochondrial localization of *PGK1*, thereby facilitating metabolic reprogramming from OXPHOS to glycolysis [4]. Accounting for the universal expression characteristics of circRNAs, these molecules have been found to be involved in the milk synthesis as well as mammary gland development of various species, such as sheep [5], cattle [6], goat [7], and rat [8]. MiRNA is a popular research topic because it can regulate gene expression in different cell types and define cell identity. Nicolas Christofoou and colleagues used miR-133a and miR-1 [9]. In vitro, human fibroblasts were reprogrammed into cardiomyocyte-like cells when combined with other small molecules; D Subramanyam et al. discovered that introducing synthetic mimics of miR-302b or miR-372 into human fibroblasts can increase the efficiency of OSKM- and OSK-mediated reprogramming of human fibroblasts [10]. MiRNA is a 21–23 nt, single-stranded, small molecule that readily synthesizes and transfects cells more easily than other small molecules. According to studies, the mRNA reprogramming system has the advantage of precise control of reprogramming factor stoichiometry compared to traditional reprogramming technology [11]. Previous studies have shown that linc/circRNAs can function as miRNA sponges to reduce miRNA activity to regulate its targets [12,13]. We propose that linc/circRNAs may serve as sponges to counteract the activities of MEF-high miRNAs during reprogramming. Many genes targeted by these miRNAs were up-regulated, and long intergenic non-coding RNAs (lincRNAs) and circular RNAs (circRNAs), which have complementary binding sites to these miRNAs, were highly expressed, indicating that lincRNAs and circRNAs may function as ceRNAs. Intriguingly, knockdown of linc/circRNAs that sponge the miRNAs, targeting OCT4 down-regulated exogenous OCT4, decreased the reprogramming efficiency, and resulted in low-grade iPSCs. Our results suggest that the ceRNA network plays an important role in cellular reprogramming. A competition mechanism called the ceRNA regulatory network is inside the ncRNA mentioned above. MiRNA can compete endogenously to remove the expression of the regulator gene. CircRNA has also been proven to remove the expression of regulator genes as a sponge of miRNA. Reprogramming involves the upregulation of pluripotent gene expression and the downregulation of differential gene expression. Some studies have shown that knocking down the lnc/circRNA of sponge-like miRNA can target and down-regulate OCT4, reduce reprogramming efficiency, and lead to low-grade iPSC [14]. The introduction of EMT at the early stage of reprogramming can effectively improve the efficiency of reprogramming, and ceRNA has been proven to be an internal part of the EMT regulation circuit [15]. These results indicate that the role of the ceRNA regulatory network in reprogramming overproduction cannot be ignored.

Here, we analyzed existing data on CiMECs; previously, we demonstrated that a combination of five small molecules (BFRTV, which includes TTNPB (B), Forskolin (F), Repsox (R), Tranylcypromine (T), and VPA (V)) can efficiently reprogram goat ear fibroblasts (GEFs) into chemically induced mammary epithelial cells (CiMECs) with a lactation function [16]. We explored the differences in the transcription profile using whole-transcriptome sequencing for the first time and analyzed the interaction between mRNA, miRNA, and circRNA. In addition, the ceRNA network was created to identify the main factors involved in the direct lineage reprogramming of somatic cells. This study will contribute to developing new ideas for studying the reprogramming mechanism and novel treatment concepts.

## 2. Materials and Methods

### 2.1. Identification of circRNA

The transcriptomic data of goat ear margin fibroblasts (GEFs) reprogrammed into mammary gland epithelial cells (CiMECs) induced by the combination of BFRTV small molecules, which were chemically obtained in our laboratory, was analyzed [16]. Using the original off-machine data (Raw Reads) obtained by Illumina sequencing, data processing is completed through processes such as removing low-quality sequences, removing adapter contamination, and removing rRNA to obtain high-quality sequences (Clean Reads). All subsequent analyses are based on clean reads. According to the splicing site information of the circRNA and the relative position of the gene structure, the circRNAs that were identified have already been systematically categorized into six distinct types, each with its own defining characteristics: classic, alter exon, intron, overlap exon, antisense, and intergenic. Subsequently, the number of exons and the length of a single exon in the classic circRNA were analyzed.

### 2.2. Prediction of circRNA Targeting Relationship

Use miranda (3.3a) to predict the targets of circRNAs of classic and antisense types and obtain their related information, and use cytoscape (3.7.1) to visualize the obtained interaction data.

### 2.3. Differential Expression Analysis of circRNA

The normalization method of SRPBM (Spliced Reads per Billion Mapping) was used to quantify the expression of circRNA, DEseq2 was used to analyze the differential expression of circRNA, and |log2 fold change| > 1.5849 and *p*-value < 0.05 were selected as the screening conditions to obtain a significant differentially expressed circRNAs and their down-regulated numbers. The expression profile analysis and the functional analysis of the differentially expressed circRNA are distinguishable, and a significant difference was observed in statistics between CiMEC and GEF circRNA before and after reprogramming. Using |fold change| > 1.5849 and *p*-value < 0.05 as screening conditions using volcano plot.

### 2.4. Functional Enrichment and Analysis of Differentially Expressed circRNA-Derived Genes Construction of PPI Network

GO and KEGG functional enrichment analyses were performed on the source genes of differentially expressed circRNAs before and after reprogramming. Then, STRING (https://cn.string-db.org/, accessed on 1 May 2023) analysis was used to establish the PPI protein interaction network between source genes.

## 3. Results

### 3.1. Sequencing and Characterization of circRNAs

**Figure 1:**
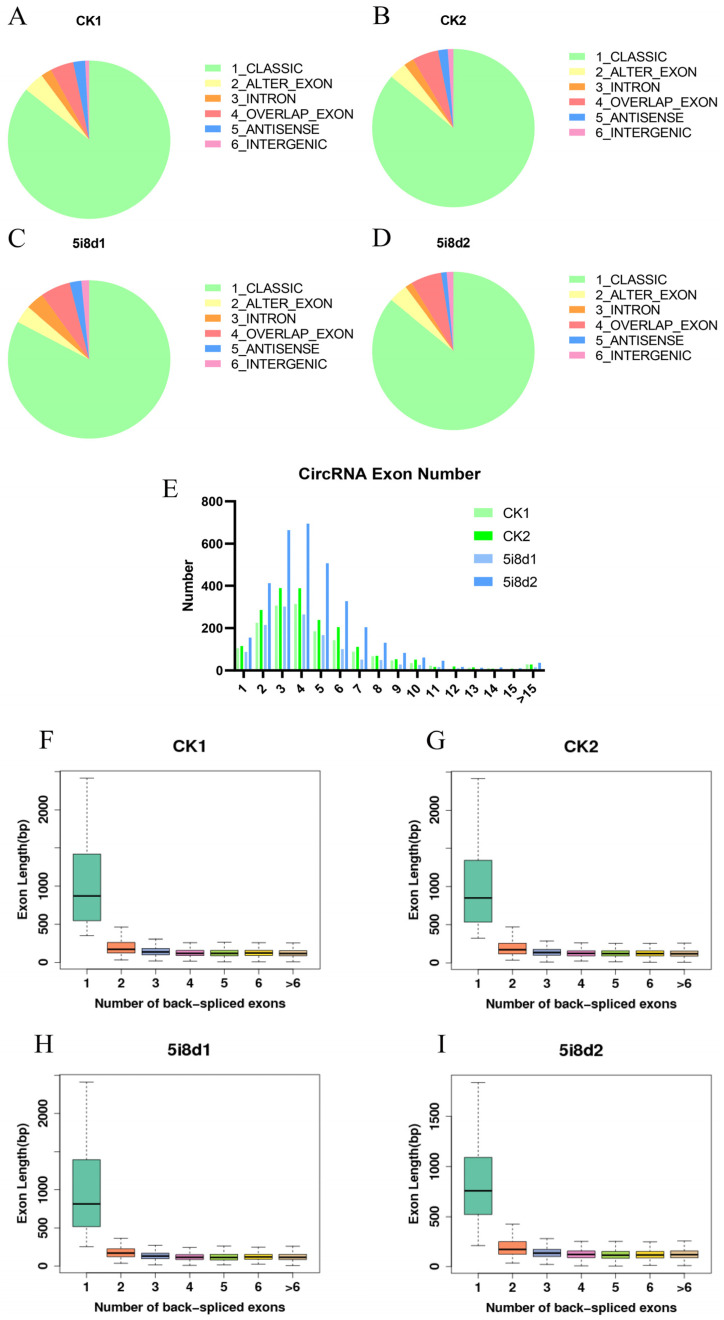
Identification of circRNAs in mammary epithelial cells of goat. (**A**–**E**) Control groups (GEF1, GEF2) and experimental groups (CiMEC1, CiMEC2) exhibited similar circRNA distribution and percentages (**E**) circRNAs exon number (**F**–**I**) Exon the length of circRNAs.

This study examined the reprogramming of the circRNAs of goat ear fibroblasts (GEFs) into mammary epithelial cells(CiMECs). CircRNAs were identified in different functional areas and classified according to six ways: classic, alter exon, intron, overlap exon, antisense, and intergenic. The total effective percentages of all functional areas of circRNA were 82.9% from the classic region, 3.6% from the alter exon region, 3.6% from the intronic region, 6.0% from the overlapped exonic region, 2.3% from the antisense region, and 1.5% from the intergenic region. Our data showed that the control groups (GEF1, GEF2) and the experimental groups (CiMEC1, CiMEC2) exhibited similar circRNA distribution and percentages (Figure 1A–D), while the majority of circRNAs of four experimental groups were between two and six exons (Figure 1E).

Among the many types of circRNAs, we selected classic circRNAs whose splicing sites were just on the exon boundary for analysis. We selected classic circRNAs for the prediction of many other types of circRNAs. Then, we determined the number of exons and the length of a single exon, and it was found that when the circRNA consisted of one exon, the length of the exon was significantly longer than that of the exons in the circRNA composed of multiple exons (Figure 1F–I).

### 3.2. The Potential Functions of the Detected circRNA’s Sponge with miRNA

**Figure 2:**
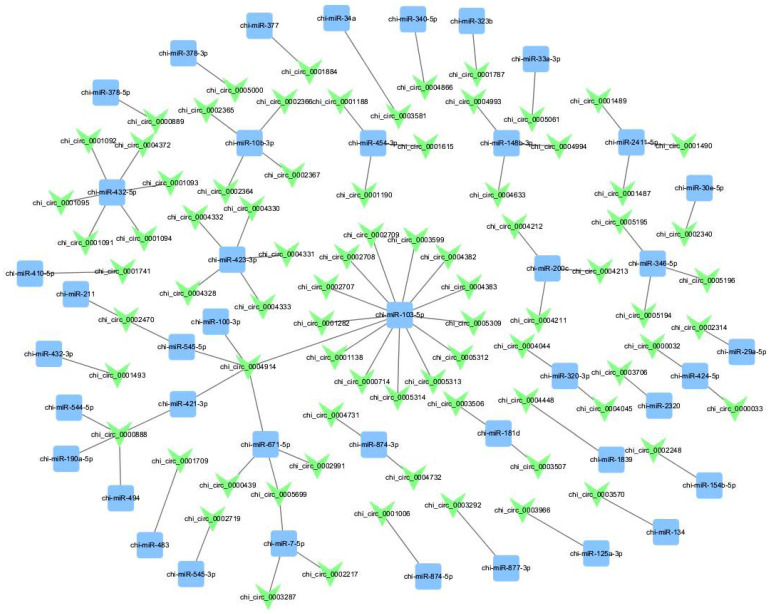
circRNA sponge miRNA visualized using cytoscape (3.7.1).

To predict the potential relation of circRNA to miRNA, we used the miranda database for target prediction on the filtered 42 miRNAs sponges by circRNAs, and circRNA-sponge miRNAs were obtained, which were visualized using a cystoscope (Figure 2). Displayofprediction results that chi_circ_0004914 targets 5 miRNAs at the same (chi-miR-100-3p, chi-miR-545-5p, chi-miR-421-3p, chi-miR-671-5p, chi-miR-103-5p); chi_circ_0000888 simultaneously sponge 4 miRNAs (chi-miR-421-3p, chi-miR-544-5p, chi-miR-190a-5p, chi-miR-494); chi_circ_0002470 simultaneously sponge 2 miRNAs, namely (chi-miR-211 and chi-miR-545-5p); and chi_circ_0005699 simultaneously sponge two miRNAs, namely (chi-miR-671-5p and chi-miR-7-5p); at the same time chi- miR-103-5p was simultaneously sponge by 14 circRNA’s. It is predicted that some of the miRNAs have a major role in mammary development. Chi-miR-200c, these miRNAs are involved in milk fat metabolism and are highly expressed in lactating than non-lactating mammary glands. Chi-miR-378-3p is involved in the lactation of dairy goat mammary glands and regulates the expression of several lipogenic enzymes in numerous tissues involving the mammary gland. Chi-miR-30e-5p regulates fatty acid metabolism in goat mammary epithelial cells. Chi-miR-423-3p has regulatory functions in mammary gland biology. Chi-miR-103-5p controls milk fat accumulation in the goat mammary gland during lactation and regulates the milk fat synthesis in goat mammary epithelial cells. The results clearly show that there was a significant difference between classic circle-RNA and the miRNA that were maybe sponge detected. Furthermore, all of the miRNAs described here are related to lactation; therefore, the conclusion suggests that these circRNAs may play a role in reprogramming cells to achieve lactation function.

### 3.3. Differentially Expressed circRNA

**Figure 3:**
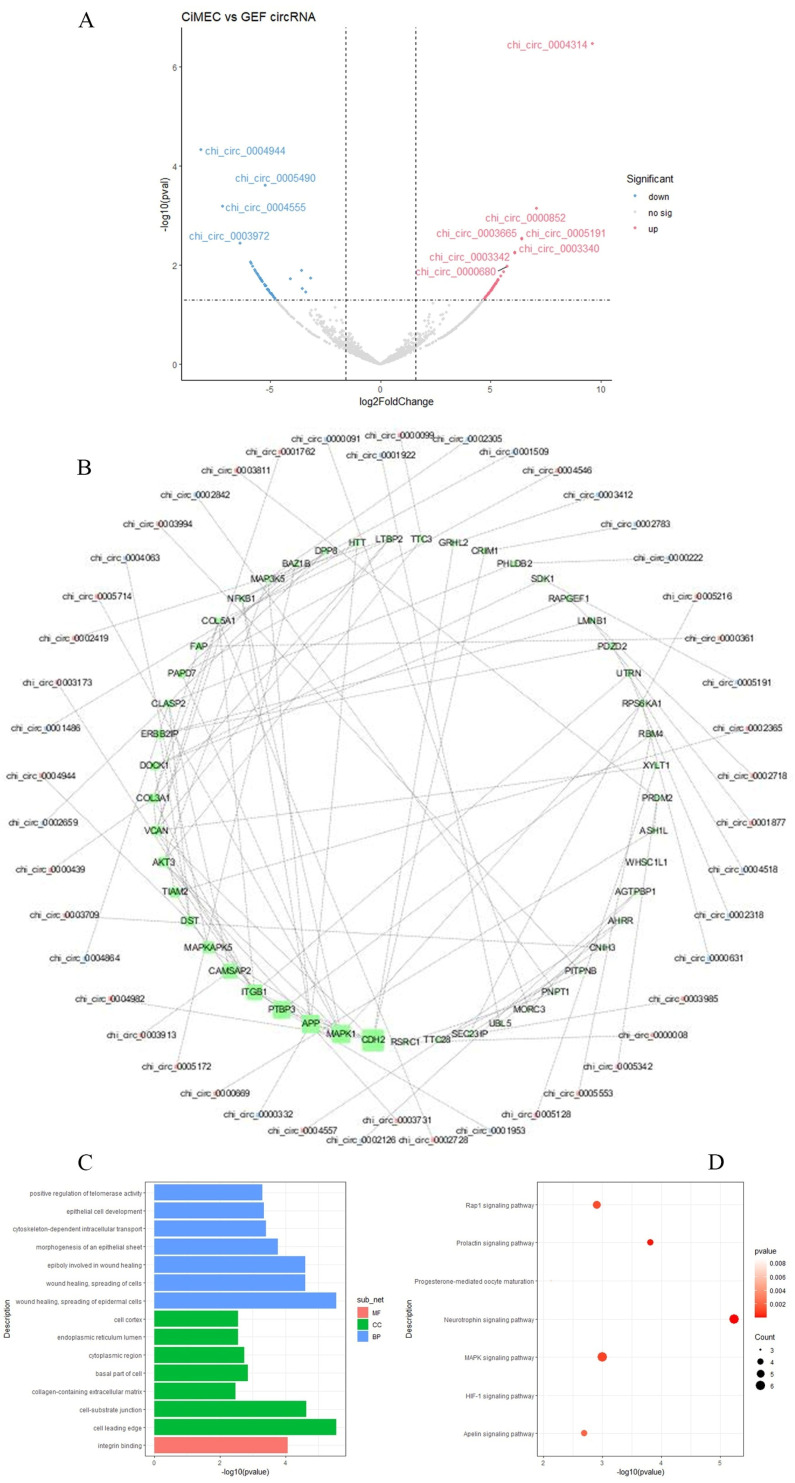
(**A**) indicates goat mammary epithelial cells have a significant amount of circRNA in the CiMEC and GEF circRNA groups. Moreover, 52 upregulates and 55 down-regulates. (**B**) PPI network construction and detection of hub genes used string to build PPI interaction network for these 95 mRNAs (**C**,**D**). Enrichment analyses results of the DEGs from the network functional enrichment analysis of GO and KEGG signaling pathways.

A total of 107 DE circRNAs from them (52 up-regulated and 55 down-regulated) were identified (Figure 3A). Chi_circ_0000852, chi_circ_0003665, chi_circ_0005191, chi_circ_0003340, chi_circ_0000680, and chi_circ_0004314 were up-regulated, respectively, while fold change of the top 20 genes in the down-regulated genes were all within five times above, chi_circ_0004944, chi_circ_0005490, chi_circ_0004555, chi_circ_0003972 all of them were down-regulated. To better study the functions of differentially expressed circRNAs, the host mRNAs were studied, and it was found that these 107 differential circRNAs were derived from 95 mRNAs.

String was used to build a protein–protein interaction network for these 95 mRNAs. Interestingly, *CDH2*, *MAPK1*, *APP*, *PTBP3*, and *ITGB1* are the core genes in this network, and some other genes can be visualized in the relationship between the circRNA and mRNA (Figure 3B). *CHD2* is the host gene for chi_circ_0004982 and encodes glycoproteins as well as calcium-dependent cell adhesion molecules. The host gene for chi_circ_0001953 is *PTBP3,* which regulates Cell proliferation, differentiation, and migration. Chi_circ_0004063 occupies a spot in the *MAPKAPK5* gene, and *MAP3K5* is highly abundant in an apoptotic pathway that plays an important role in the development and function of the mammary gland. All these genes were upregulated, while the host gene of chi_circ_0005714, *MAPK1*, controls the synthesis of milk proteins. *APP* is the chi_circ_0005714 host gene. Interactions between proteins regulate cell motility and transcription.

Based on the prediction that the functions of circRNA and host genes are known, we analyzed the pathways enrichment of the host genes producing DE-circRNAs to predict their potential functions. Then, we performed functional enrichment analysis of GO and KEGG signaling pathways; interestingly, we found that biological processes related to epidermal cell spreading (GO:0035313), morphogenesis of epithelial sheets (GO:0002011), epithelial cell development (GO:0002064), which are related to the development process of mammary epithelial cells; extracellular matrix containing collagen (GO:0062023), cell cortex (GO:0005938), etc., are also found in the cell components in the reprogramming of epithelial cells part of the process (Figure 3C). KEGG enrichment shows that before and after the reprogramming process, the source mRNA of the differential circRNA is related to hormone-related signaling pathways such as prolactin signaling pathway and progesterone-mediated oocyte maturation, and is related to the MAPK signaling pathway in the reprogramming process. It is related to the HIF-1 signaling pathway of cell proliferation and the apelin signaling pathway of anti-inflammation and anti-oxidation (Figure 3D). These results indicate that circRNA may play a key role in mammary epithelial maintenance in reprogramming cells.

### 3.4. Prediction of Differentially Expressed circRNA–miRNA–mRNA

**Figure 4:**
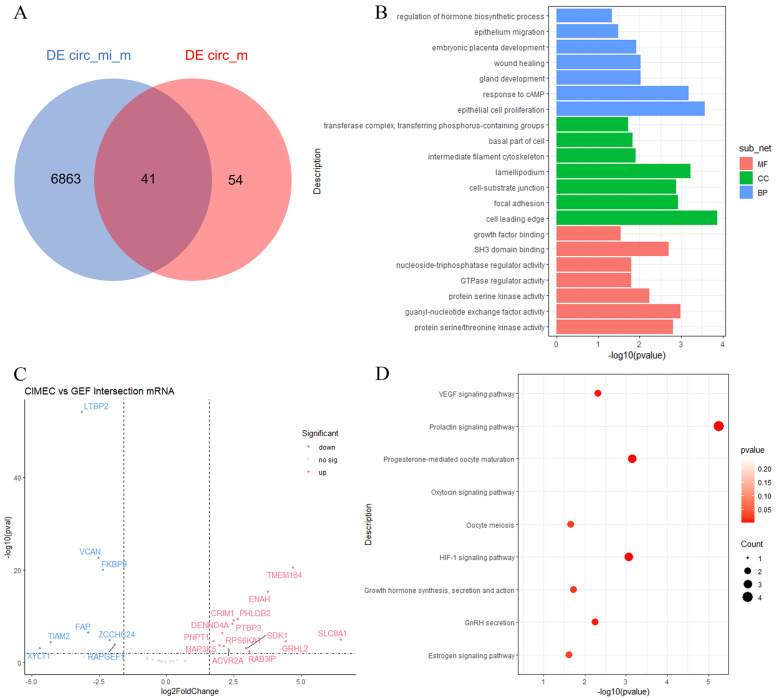
Prediction of DE circRNA–miRNA–mRNA (**A**) CircRNA localization in mammary epithelial cells of goat between DE circRNA_miRNA_mRNA and DE circRNA. (**B**,**D**) GO and KEGG enrichment annotations of DE-circRNA host genes were performed. (**C**) CiMEC and GEF intersection of mRNA before and after reprograming.

The differentially expressed circRNA before and after reprogramming can only sponge to two miRNAs, namely miR-10b-3p and miR-671-5p. These two miRNAs were used to predict target genes using the miRanda database, and finally, 6904 predicted mRNAs were obtained. Using the intersection of the gene list obtained above and the gene list of the source mRNA of DE circRNA, 41 target genes were found to be targeted at the same time (Figure 4A), and the filtering conditions of |log2 fold change| > 3 and *p*-value < 0.05 were used to filter them. Differential expression analysis found that 22 genes changed before and after reprogramming, of which 14 genes were up-regulated and 8 genes were down-regulated (Figure 4C). A large number of studies have shown that circRNA can affect the transcription of host genes, so afterward, GO and KEGG enrichment of the host genes of DE-circRNAs was performed on 41 genes. Interestingly, we found that there were signals related to epithelial cell proliferation (GO: 0050673), glandular development (GO: 0048732), and regulation of hormone biosynthetic processes (GO: 0046885). From the perspective of molecular functions, host genes are mainly involved in epithelial cell proliferation and gland development. (Figure 4B). The KEGG analysis showed that these genes were related to hormone-related signaling pathways, such as prolactin signaling pathway, growth hormone synthesis, estrogen signaling pathway, and oxytocin signaling pathway (Figure 4D). These results indicate enrichment analysis from DE-circRNAs, suggesting that these DE-circRNAs may have potential regulatory mechanisms for mammary gland development and lactation.

### 3.5. Establishment of ceRNA Network

**Figure 5:**
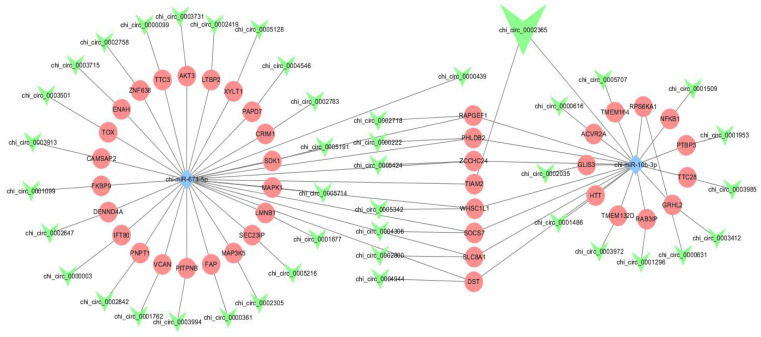
Competative endogenous RNA (ceRNA) networks in which triangle shape in green color shows circRNA, oval shape in pink color represents genes, diamond shape blue colour represents miRNA.

Based on the above analysis and prediction, we discovered and established a co-expression network between DE circRNA–miRNA–mRNA and DE circRNA-mRNA. Finally, we used 41 mRNAs, 42 DE-circRNAs, and 2 miRNAs to construct a competitive endogenous network (Figure 5). We found that circ_0002365 was also derived from *TIAM2* in sponge miR-10b-3p. In the competing network between *TIAM2* and circRNA, miRNA inhibits this gene and maintains epithelial function. It is interesting that most of the 41 target genes are related to lactation and epithelial cell development. Among the key candidates of circRNAs that have the binding ability, with the adsorption of miR-10b-3p, chi_circ_0000222 up-regulates *PHLDB2*, chi_circ_0000616 up-regulates *ACVR2A*, chi_circ_0001486 up-regulates *HTT*, chi_circ_0001509 up-regulates *NFKB1*, chi_circ_0003412 up-regulates *GRHL2*, chi_circ_0002035 down-regulated *GLIS3*, respectively. While with the adsorption of chimiR-671-5p, chi_circ_0002305 up-regulates *MAP3K5*, chi_circ_0003731 down-regulates *AKT3*, chi_circ_0005714 down-regulates *MAPK1*. Through the establishment of the cerNA network, we predicted several genes that were related to mammary gland development, mammary epithelial cells, and hormonal regulation.

## 4. Discussion

Goats are not only an important source of dairy products but also an ideal small animal model to study the transcriptome and expression characteristics of the mammary epithelial cells. Circular RNAs (circRNAs) are a type of non-coding RNA that playsa crucial biological role in the development of mammary glands and breastfeeding in mammals, functioning as “miRNA sponges” by adhering to proteins and triggering a number of biological processes [17]. In recent years, as a novel non-coding RNA, circRNA has emerged as a new research topic. Through the rapid development of high-throughput genome sequencing and computational biology, investigators revealed that circRNAs are prominent in a number of species and have important biological functions [18]. Here, we have generated the CiMECs and systematically explored the differences in the transcription profile for the first time using whole-transcriptome sequencing and analyzed the interaction among mRNA–miRNA–lncRNA and circRNA. Furthermore, the ceRNA network was constructed to identify the key factors involved in somatic cells’ direct lineage reprogramming. Based on our findings, circRNAs found in our study may be able to operate as a regulator of milk synthesis and improve the growth and development of mammary glands and mammary epithelial cells.

### 4.1. Establishment of ceRNA Networks for Candidate circRNA’s

As an exceptionally competitive endogenous RNA, circRNA is abundant in miRNA-binding sites and has the ability to adsorb mRNAs via the “miRNA sponge” effect. Then, it eliminates the restriction of mRNAs and, therefore, indirectly regulates gene expression. This observation shows that circRNAs may work as ceRNAs and that both circRNAs and mRNAs can target miRNAs. In this study, we analyzed DE-circRNAs which were identified through functional enrichment analysis, and hypothesized the regulatory role of these DE-circRNAs via the circRNAmiRNAmRNA interaction regulatory network.

Mammary gland development and functional activation of lactation are tightly regulated by hormones, growth factors, and nutrient availability [19]. Previous research revealed that circRNA absorbs miRNA and thus facilitates the regulation of the biosynthesis of unsaturated fatty acids via bovine mammary epithelial cells [20,21], while in another study, it was revealed that circHIPK3 stimulates the growth of cow mammary epithelial cells [22]. CircRNAs were found to be important for the production of milk in addition to the development of the mammary glands in a variety of species, including sheep [5]. In cattle, circRNAs act as sponges for miR-2284, which regulates casein gene translation and milk protein synthesis [6]. Our results for circRNA–miRNA–mRNA interaction regulatory networking revealed that reprogramming of CiMECs led to the absorption of the mRNAs through the miRNA sponge effect and regulated several genes having beneficial roles in lactation, milk synthesis and development of mammary epithelial cells and/or gland. While, with the adsorption of mRNAs through the effect of chimiR-671-5p sponge, chi_circ_0002305 regulated *MAP3K5*. In our study, most predicted target genes were almost related to mammary gland development, mammary epithelial cells, and milk production, such as *NFKB1*, *MAP3K5*, *AKT3*, and *MAPK1*. *PHLDB2* plays a crucial role in the regulation of epithelial-to-mesenchymal transition [23]. *HTT* and *MAPK1* genes contribute to the regulation of mammary stem cell proliferation differentiation [24,25] and maintaining epithelial polarity [26]. In bovine mammary epithelial cells, *NFKB1* plays a crucial role in mammary gland morphogenesis [27] and transcriptional activation of milk synthesis [28]. *MAP3K5* and *MAPK1* were found to function in mammary gland development, regulating milk protein synthesis and lactation [29,30,31]. *AKT3* plays a role in the development of the mammary gland as well as lactation [32,33]. *AKT3* genes that regulate kinase and *ATP* binding function may have an enormous effect on the molecular processes associated with milk production [34]. The expression of *GRHL2* plays a significant role in the regulation of cell adhesion in luminal epithelial cells [35]. The key gene *RAPGEF1* has been found to be associated with lactation persistence [36]. Some of the miRNAs that were sponged by the key circRNAs, e.g., chi-miR-200c, actively participate in the process of the metabolism of the milk fat [25], while in some studies, chi-miR-200c increased the levels of expression in lactating mammary glands compared to non-lactating ones [37]. Chi-miR-200c plays an important role in human adipocyte cells, pertaining to the metabolic process of lipids [38]. In previous studies, chi-miR-10b-3p relates to high levels of expression in goat ovaries [39]. Moreover, chi-miR-30e-5p in previous studies regulates the synthesis of fatty acids in goat mammary epithelial cells [40]. Chi-miR-103-5p during lactation regulates the accumulation of milk fat in the goat mammary gland [41]. Chi-miR-7-5p was found to have a key role in increased levels of expression in the pituitary gland [42]. Here, we construct a network to promote lactation and maintain epithelial character.

### 4.2. Identification and Detection of Key Candidate circRNA’s

The host genes of the DE-circRNA mainly participated in the GO terms and KEEG pathways related to mammary epithelial cell proliferation and development, regulation of hormones related to mammary gland development and milk production, and lactation. The major enriched KEEG pathways were the HIF-1 signaling pathway, prolactin signaling pathway, progesterone-mediated oocyte maturation, GnRH secretion, estrogen signaling pathway, and oxytocin signaling pathway. To better regulate milk protein gene expression and mammary epithelial cell development, HIF-1 is a heterodimeric transcription factor that increases phosphorylation of signal transducer and activator of transcription 5A(STAT5A) in mammary epithelial cells [43,44]. Prolactin is essential for the development of the mammary gland throughout late pregnancy and the early postpartum period as well as the production of the carpus lutetium after blastocyst implantation [45,46]. During pregnancy, prolactin plays a role in both breast proliferation and differentiation, in addition to its role as a crucial regulator of the mammalian reproductive process [47]. Progesterone, either by itself or in combination with prolactin, has been shown in previous research to play a role in the development of milk alveolar cells, which are responsible for the secretion of milk during lactation [48]. Progesterone pathways regulate meiosis, fertilization, embryonic development, and implantation in the ovarian tissue and feto-maternal unit [49]. The function of the MAPK signal pathway is mainly found in the process of branching morphological development in the submandibular gland [50] and mammary gland development [51]. Early development and enlargement of the ducts into the mammary fat pad, as well as a decrease in milk production during pregnancy, leads to colostrum production, depending on estrogen binding to its receptor ER [47,52]. Oxytocin is well-known for its effect of inducing myoepithelial cell contraction and, therefore, milk ejection. Oxytocin is a key hormone in nursing and in regulating milk production [53]. The enriched host genes of DE-circRNA in the HIF-1 signaling pathway, prolactin signaling pathway, progesterone-mediated oocyte maturation, GnRH secretion, estrogen signaling pathway, and oxytocin signaling pathway here in this study imply that the potential function of circle-RNA is related to the maintenance of CiMEC’s identity.

## 5. Conclusions

The aim of the present research was to examine the identification and expression of circRNAs in mammary epithelial cells before and after reprogramming into two groups. A total of 107 DE circRNAs (52 up-regulated and 55 down-regulated) were identified in CiMECs. Among those, several DE circRNAs participated in GO terms and KEEG pathways related to mammary epithelial cells’ growth and development. The endogenous competition revealed that we are the first to construct the expression profile of circle RNA in large domestic animals with somatic cell reprogramming. We found that circle RNA after reprogramming may be involved in the maintenance of breast epithelial identity and function of reprogrammed cells, and the reprogramming process has a relationship with CiMECs.

## Data Availability

All data generated or analyses during this study are included in this published article, “Restoring mammary gland structures and functions with autogenous cell therapy”. Biomaterials, 2021. 277: p. 121075. (Zhang et al., 2021) [16], accession code GSE142551.
